# Inverse Filtering for Frequency Identification of Bridges Using Smartphones in Passing Vehicles: Fundamental Developments and Laboratory Verifications

**DOI:** 10.3390/s20041190

**Published:** 2020-02-21

**Authors:** Nima Shirzad-Ghaleroudkhani, Mustafa Gül

**Affiliations:** Department of Civil and Environmental Engineering, University of Alberta, Edmonton, AB T6G 1H9, Canada; shirzadg@ualberta.ca

**Keywords:** inverse filter, bridge monitoring, smartphone, vehicle–bridge interaction, frequency spectrum

## Abstract

This paper puts forward a novel methodology of employing inverse filtering technique to extract bridge features from acceleration signals recorded on passing vehicles using smartphones. Since the vibration of a vehicle moving on a bridge will be affected by various features related to the vehicle, such as suspension and speed, this study focuses on filtering out these effects to extract bridge frequencies. Hence, an inverse filter is designed by employing the spectrum of vibration data of the vehicle when moving off the bridge to form a filter that will remove the car-related frequency content. Later, when the same car is moving on the bridge, this filter is applied to the spectrum of recorded data to suppress the car-related frequencies and amplify the bridge-related frequencies. The effectiveness of the proposed methodology is evaluated with experiments using a custom-built robot car as the vehicle moving over a lab-scale simply supported bridge. Nine combinations of speed and suspension stiffness of the car have been considered to investigate the robustness of the proposed methodology against car features. The results demonstrate that the inverse filtering method offers significant promise for identifying the fundamental frequency of the bridge. Since this approach considers each data source separately and designs a unique filter for each data collection device within each car, it is robust against device and car features.

## 1. Introduction

Recent technology developments have provided a great opportunity for efficient urban management in order to overcome the challenges they face today. In this context, Smart City is a concept to develop a city where smart sensing, computing, and communication technologies are employed to improve the efficiency of the critical infrastructure components and services of a city [1,2,3,4]. Sustainability of a smart city is intertwined with its transportation infrastructure [5]. Hence, many studies have focused on the applications of smart technologies in transportation infrastructure [6,7].

In a developed transportation network, bridges are key components and their dysfunctionality results in major disruptions in the network [8,9]. A considerable portion of bridge structures in modern countries have reached their design life and need to be monitored and retrofitted to remain in service. For instance, Canadian Infrastructural Report Card [10] has estimated about 26% of bridges in Canada to be in ‘fair’, ‘poor’, or ‘very poor’ condition. These bridges are under the risk of potential deterioration and their stable structural performance relies on appropriate monitoring and maintenance operations. To this end, Structural Health Monitoring (SHM) techniques have been proposed and studied in the literature [11].

Most conventional SHM methods use fixed sensors to collect data from bridges, such as [12,13,14,15]. Although the efficiency of these methods has been proven in many studies, employing such direct SHM methods to a large number of bridges may not be feasible. Instrumentation of each bridge with fixed sensors and creating a data collection network is costly and time-consuming, and cannot be employed to all bridges in a wide metropolitan area. Hence, indirect bridge monitoring methods [16] have been proposed through focusing on sensors placed in passing vehicles as moving sensors, which makes it possible to monitor a large number of bridges at a global level without installing fixed sensors.

Indirect bridge monitoring concept was first proposed by Yang et al. [17] where the dynamic analysis of vehicle–bridge interaction was performed for a simple 2D beam model as the bridge and a moving mass-spring system as the vehicle. In that paper, they successfully extracted bridge frequency from the vibration of the vehicle. Later on, many studies followed the path of indirect bridge monitoring, which can be divided into analytical/numerical analyses [18,19,20,21], lab-scale experiments [22,23,24], and real-life experiments [25,26]. These studies support the fact that the vibration of the passing vehicle contains bridge dynamic response which can be extracted to evaluate bridge condition. Although using dedicated sensors in vehicles provides valuable means of effective real-time monitoring of the population of bridges within a city, an alternative way of employing indirect monitoring methods using the smartphones of the passengers in the vehicles has also been proposed recently as discussed in the following.

In the context of smart cities, smartphones equipped with many sensors—such as accelerometer, gyroscope, GPS, etc.—are critical devices as they may provide valuable data. The high popularity of smartphones makes it possible to create a dense data source through crowdsourcing methods. For instance, smartphones have been employed as effective tools to estimate traffic incidents, like congestions or accidents [27,28] or road conditions [29]. Recently, the application of smartphones in indirect health monitoring has been the subject of many studies. Mei and Gül [30] used a smartphone to detect multiple damage states on a lab-scale bridge model through an indirect damage detection method. In another study, the possibility of capturing bridge frequencies using smartphones was investigated by Matarazzo et al. [31] in a real-life experiment. In another study, authors [32] investigated the robustness of indirect monitoring methods against vehicle features, such as suspension stiffness and speed, by performing a lab-scale experiment. All these studies note that the vibration of the vehicle is more dominated by vehicle features—such as suspension, mass, speed, etc.—than bridge frequencies.

Signal processing and filtering methods have been widely used in SHM and bridge engineering. For instance, Ding et al. [33] employed an adaptive finite impulse response filter to study temperature effects on bridges. In addition, using Kalman filter for bridge health monitoring was a subject of many studies [34,35]. However, employing an appropriate filtering method in indirect bridge monitoring is a challenge since every signal is recorded on a different device located on a different vehicle. Thus, any effective filter resulting in bridge features when applied to the vibration data of the moving vehicle needs to be unique to the vehicle and the device. This paper proposes inverse filtering method to achieve this goal.

In general, inverse filters are designed to extract a specific feature from a noisy signal. In other words, the signals in noisy conditions are used to create a filter which then can be applied to other signals to remove the effect of the noise. Some of the first applications of inverse filtering were in voice processing [36,37]. Later, it was used in other fields such as image processing in medical sciences [38] and geophysics [39]. This study proposes an inverse filtering method for indirect monitoring of bridges, which utilizes the vibration data recorded on the vehicle while moving off-bridge, i.e., moving on the ground, to filter out car features from the vibration of the vehicle while moving on-bridge. Therefore, each device on each vehicle will have a unique filter which is compatible with the features of the device and the car, and makes it possible to be applied to population of devices to monitor bridges. For simplicity, the proposed methodology considers similar conditions for off- and on-bridge tests, including surface roughness, mass and speed of the vehicle, orientation of the smartphone, etc. In the following, first the methodology of the proposed signal processing and inverse filtering is explained. Later, the experiment setup is demonstrated. Finally, the analysis results are discussed.

## 2. Methodology

In this section, first, the fundamental idea of vehicle–bridge interaction and its application to bridge health monitoring is discussed. Afterward, the frequency-domain analysis applied to the time-domain vibration signals is described. Subsequently, the process of designing and applying inverse filtering technique is presented.

### 2.1. Vehicle–Bridge Interaction

When a vehicle is moving over a bridge, vibrations of vehicle and bridge are coupled due to the dynamic interaction. In other words, the vibration recorded on the vehicle includes both the vehicle and the bridge features. One of the earliest studies to investigate this concept was Yang and Yau [40]. They considered a simple 2D beam model as the bridge and a moving spring-mass system as the vehicle. They derived the following coupled formulation

(1) $$\{\begin{array}{c} \bar{m}(x)u_{b}^{\prime \prime }(x,t)+E(x)I(x)u_{b}^{\prime \prime \prime }(x,t)=c(x,t)\\ m_{v}u_{v}^{\prime \prime }(t)+k_{v}\left[u_{v}(t)-u_{b}(x,t)\right]=0 \end{array}$$

In Equation (1), vehicle-related parameters include *x* as the distance of the vehicle to the bridge end, *v* as the speed of the vehicle, *m_v_* and *k_v_* as the mass and stiffness of the vehicle, and *u_v_* as the vertical displacement of the vehicle relative to the initial position. Bridge-related parameters are $\bar{m}$ as the distributed mass of the bridge per unit length, *E* and *I* as the elastic modulus and moment of inertia of the bridge section, respectively, and *u_b_* as the vertical displacement of the bridge relative to its equilibrium position. In addition, *t* denotes the time, *c* represents the contact force, and prime notation shows the time-derivative function. Actually, the first equation represents the governing dynamic equilibrium of the bridge, and the second one is of the vehicle. Equation (2) demonstrates that the dynamic response of the vehicle is coupled with and include dynamic features of the bridge. Therefore, it is possible to extract bridge characteristics from vehicle response using appropriate signal processing techniques. Based on this fact, this study proposes a novel filtering method in indirect health monitoring of bridges to extract the fundamental frequency of the bridge using acceleration response of the vehicle. Next section focuses on the first step of the methodology, i.e., converting time-domain acceleration signal to frequency-domain spectrum.

### 2.2. Frequency-Domain Analysis

In this research, in order to change time-domain acceleration signals to frequency-domain spectrums, averaged discrete Fourier transform (ADFT) is applied. In this method, instead of calculating the Fourier transform of the whole signal, small windows of the signal are transformed separately and then the average of resulting spectrums is calculated. In order to explain ADFT in more details, first consider discrete Fourier transform (DFT) equation as

(2) $$X[k]=\overset{N-1}{\underset{n=0}{\sum }}a[n]e^{-j\frac{2\pi }{N}kn}$$

in which *a* is the acceleration signal, *N* is the number of data points in the signal, and *X* denotes the vector of DFTs, i.e., a complex vector containing amplitude and phase values. Also, index *k* represents step frequencies through the equation

(3) $$f_{k}=\frac{k}{N}f_{s}\;\;\mathrm{for}\;0\leq k\leq N-1$$

where *f_s_* is the sampling frequency of the acceleration signal. Applying Equation (2) to the entire acceleration signal leads to an extremely fluctuated spectrum, which is not suitable for filter design. On the other hand, if the signal is divided into multiple windows and DFT process is separately applied to each window, followed by averaging all the spectrums, the resulting spectrum will be smoother. These segments are selected through applying a window function to the raw signal. A plethora of window functions is proposed for selecting sub-signals in the literature [41]. Here, the Hamming window is considered as follows

(4) $$w[n]=0.54+0.46\cos\frac{2\pi n}{N_{w}-1}\;\;\mathrm{for}\;\left|n\right|\leq \frac{N_{w}-1}{2}$$

In Equation (4), *N_w_* is the number of terms of the window, representing window length. Note that selected windows are not mutually exclusive to account for the effect of the transition resulting in an overlap between windows of the signal. Therefore, the ADFT of the signal is calculated through the equation

(5) $$\bar{X}[k]=\frac{1}{M}\overset{M}{\underset{m=1}{\sum }}\overset{N-1}{\underset{n=0}{\sum }}w_{m}[n]a[n]e^{-j\frac{2\pi }{N}kn}$$

**Figure 1:**
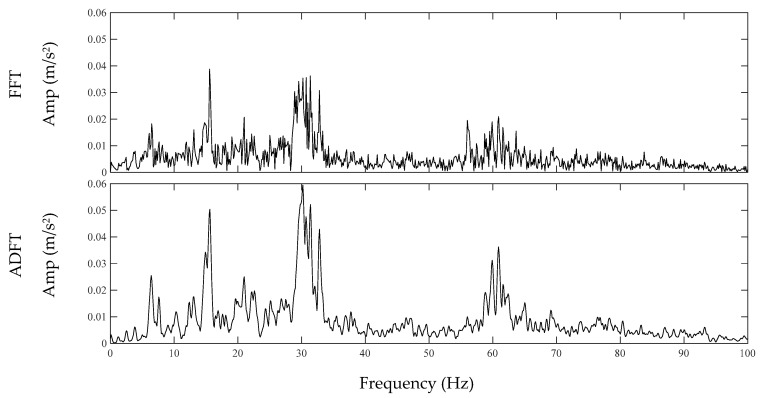
FFT vs. ADFT spectrums.

In Equation (5), *w_m_* is the function for *m*th window and *M* is the total number of windows calculated through

(6) $$M=1+\frac{N-N_{w}}{(1-p)\cdot N_{w}}$$

where *p* is the overlap percentage of the windows. Figure 1 illustrates the resulting FFT and ADFT spectrums of an acceleration signal recorded on the robot car. As seen, ADFT spectrum is smoother and more appropriate for filter design and peak analysis. The resulting ADFT spectrums are used to design the inverse filter which is explained in the next section.

### 2.3. Inverse Filter

**Figure 2:**
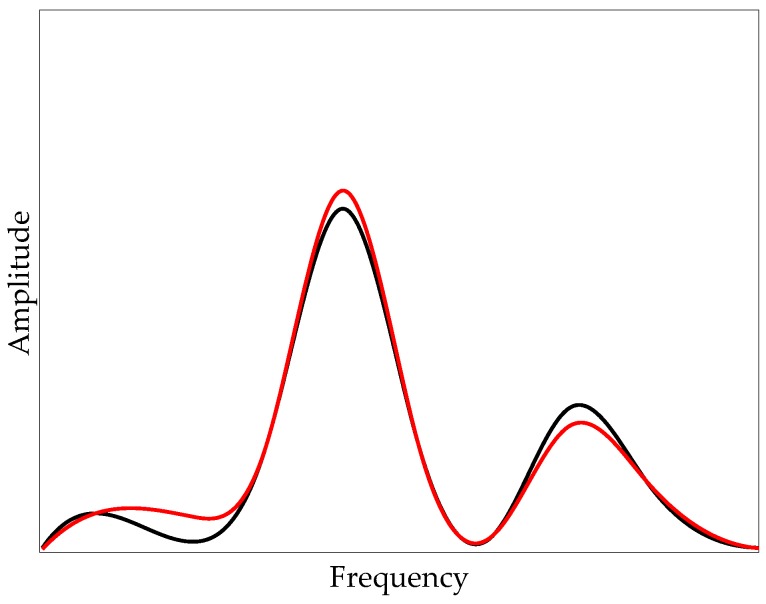
Hypothetical spectrum of off-bridge (black) and on-bridge (red) acceleration signals.

In this paper, a novel method of filtering is proposed based on the comparison of off-bridge and on-bridge acceleration signals. In fact, most of the frequency content of the recorded signals are dominated by moving car features, like speed, suspension, weight, engine vibrations, etc. These effects are present in both off-bridge and on-bridge data. However, bridge frequency is only expected to appear in the on-bridge data. Hence, if a filter is designed to remove major frequency content of the off-bridge data, and then applied to the on-bridge data, it is expected that the filtered on-bridge data will contain mostly frequencies related to the bridge dynamics. For more clarity, consider the hypothetical spectrum of the off-bridge and on-bridge signals as illustrated in Figure 2 using black and red curves, respectively. These curves are created using cubic polynomials. It is expected that the major peaks of the off-bridge spectrum will be present in the on-bridge one. However, if the frequency of the bridge is not close to the major frequencies of the car, there will be an unexpected change in the amplitude of the spectrum at a specific frequency, as seen in Figure 2. This relatively small change may not be considered as a peak in the on-bridge spectrum; while after applying the inverse filtering, this peak will become more visible.

**Figure 3:**
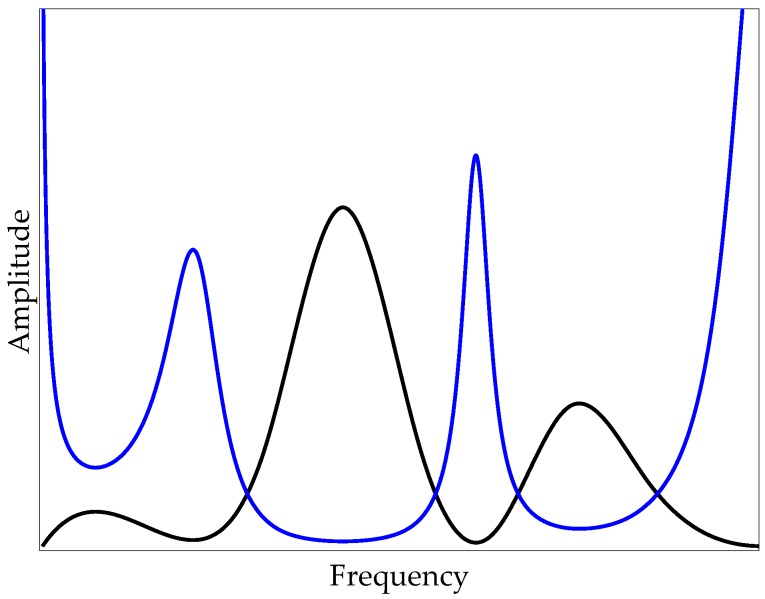
Hypothetical spectrum of the off-bridge signal (black) and corresponding filter shape prototype (blue).

In order to design the filter shape, the off-bridge spectrum is considered. As discussed in the previous section, ADFT is used to convert the time-domain off-bridge signal to the frequency domain. The proposed windowing and averaging technique ensures that a sudden external factor, such as a sharp road defect, which drastically alters the shape of the spectrum of a single window, does not affect the total shape of the resulting averaged spectrum. Afterward, the spectrum is inverted to form the filter shape prototype using the equation

(7) $$\tilde{F}[k]=\frac{1}{\bar{X}[k]}$$

where $\bar{X}$ denotes the ADFT spectrum of the off-bridge signal and $\tilde{F}$ represents filter shape prototype. In Figure 3, the black curve shows the hypothetical ADFT of the off-bridge signal, and the blue curve represents its inverse, which would be the prototype for the inverse filter. As expected, the filter shape amplifies the frequencies with low amplitude in the off-bridge spectrum and suppresses those with higher amplitudes, i.e., peaks, in the off-bridge spectrum.

In order to apply the inverse filter to the on-bridge spectrum, filter shape prototype needs to be scaled. Since filter prototype was designed based on the pure inverse function, it will scale all amplitudes to one, which is not a meaningful scale for acceleration amplitudes. Furthermore, the amplitude level of the on-bridge signal may be different than that of the off-bridge because of the external factors, like surface roughness or road defects. Here, the mean value of the spectrum as a measure of the energy level within the signal is considered for scaling, which is calculated by

(8) $$\bar{Y}=\frac{\overset{N-1}{\underset{0}{\sum }}Y[k]}{N}$$

where $\bar{Y}$ denotes the mean value of the off-bridge spectrum. Therefore, the inverse filter shape will follow this equation

(9) $$F[k]=\left(\bar{Y}\right)\tilde{F}[k]=\frac{\left(\bar{Y}\right)}{\bar{X}[k]}$$

**Figure 4:**
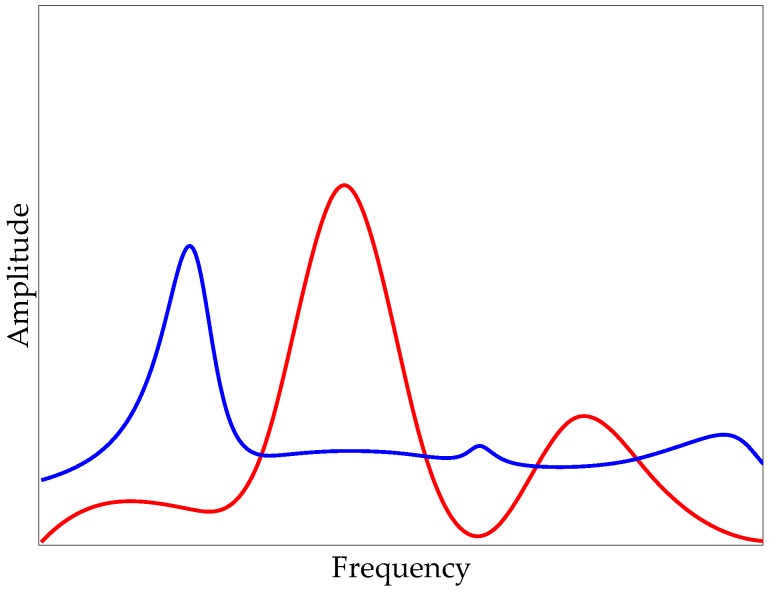
Hypothetical on-bridge unfiltered (red) and filtered (blue) spectrums.

**Figure 5:**
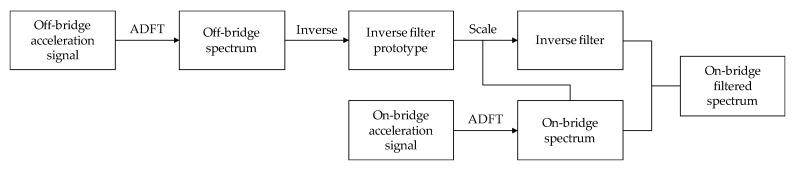
Flowchart of the proposed inverse filtering methodology.

The filter in Equation (9) can be applied to the on-bridge spectrum to form the filtered spectrum

(10) $$Y_{f}[k]=F[k]Y[k]=\frac{\left(\bar{Y}\right)}{\bar{X}[k]}Y[k]$$

in Equation (10), *Y_f_*[*k*] denotes the filtered spectrum. Figure 4 demonstrates the application of this procedure to the hypothetical spectrums in Figure 2 and Figure 3, where the red curve represents the unfiltered on-bridge spectrum and the blue curve shows the filtered spectrum. As seen, the resulting filtered spectrum has one major peak at the frequency of the deviation in amplitudes of off- and on-bridge spectrums. Although there might be some fluctuations at other frequencies, the major peak with significantly higher amplitude and prominence expresses the desired frequency of the target. The flowchart of the proposed method is presented in Figure 5. In the next section, the proposed method is applied to experimental data for verification.

## 3. Experimental Setup

In this section, the details of the experimental setup and instrumentation are introduced. First, the bridge model is described. Then, the robot-car, used as the moving vehicle, is explained. Finally, the data collection instruments employed in the experiment are presented.

### 3.1. Bridge Model

**Figure 6:**
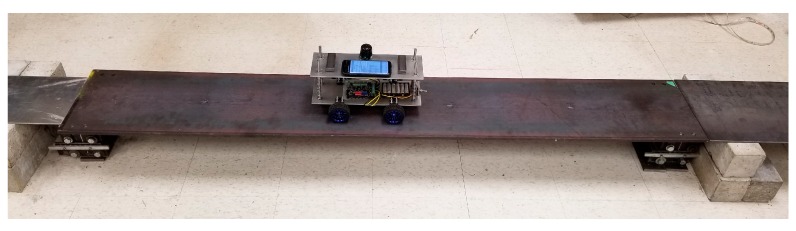
Simply supported bridge setup along with the instrumented robot vehicle.

**Figure 7:**
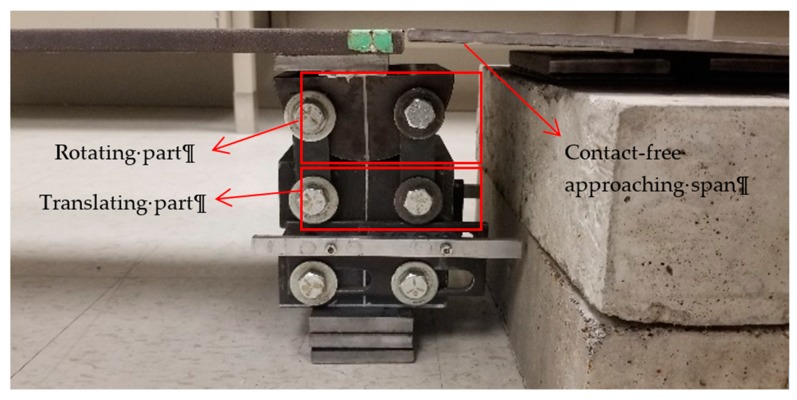
Pin-roller support setup.

In this study, a simply-supported bridge which consists of one steel plate as the deck and two supports of pin and roller is considered, as shown in Figure 6. Steel plate is hot rolled of type W44, which has the modulus of elasticity of 200 GPa, yield strength of 250 MPa, and ultimate strength of 310 MPa. The plate is 2 m long, 330 mm wide, and 12.7 mm thick. The weight of the plate is 60 kg. Here, one pin support and one roller support are employed to carry the steel deck. In Figure 7, the pin-roller support structure is illustrated. The structures of both support types are similar, except that the pin is prevented from moving horizontally while the roller is free to move. Furthermore, an approaching span is also connected to the main span, used for the first few seconds that the car accelerates and reaches the constant target speed. Likewise, another plate is used at the end of the bridge, letting the car leave the bridge at the target constant speed and then stop. An additional small thick plate is placed between the main span plate and the supports to prevent any contact of approaching or end spans with the main span and hence providing free rotation for supports.

### 3.2. Robot Car Model

**Figure 8:**
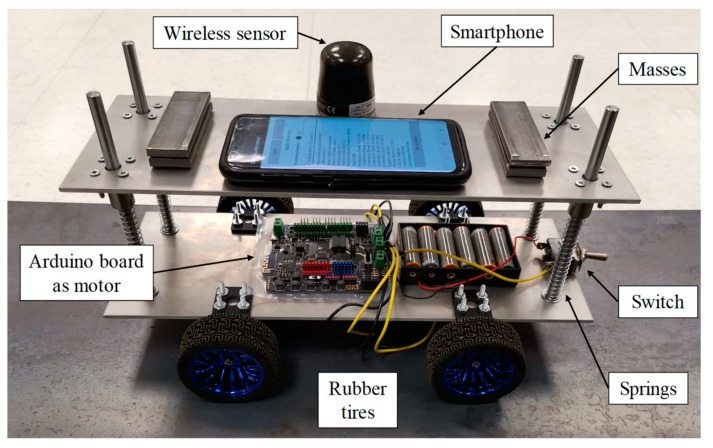
The custom-designed and -built robot car.

**Figure 9:**
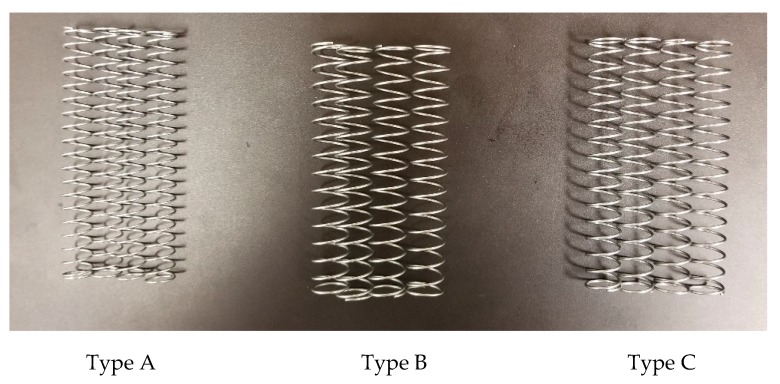
Three spring types used in the experiment.

In this study, a robot car was designed and built as shown in Figure 8. This car consists of two identical rectangular aluminum plates of 350 mm by 125 mm with a thickness of 3.1 mm. These two plates are connected to each other by four aluminum rods with the radius of 4 mm and length of 15 cm. Four springs are used together with aluminum rods to account for the suspension system of the car. In this experiment, three spring types with stiffness values of 425, 615, and 726 N/m are employed, which are herein referred to as A, B, and C, respectively, as shown in Figure 9. In addition, three different car speeds of 0.2, 0.3, and 0.4 m/s are considered in this experiment.

The motor and the wheels are connected to the bottom plate. Each wheel is powered through a separate motor, all controlled by the main board on the car. The total weight of the car is 2.3 kg, consisting of 1 kg of the top plate, including the smartphone, the wireless sensor, and added masses, and 1.3 kg of the remaining parts connected to the bottom plate. It should be noted that the 3.8% mass-ratio of car to bridge in this experiment is larger than most real-life cases, which relatively amplifies bridge vibrations. However, since usually in real-life cases there are many cars simultaneously traveling over the bridge, the amount of the induced vibration in the bridge in this experiment due to a single passing car could be comparable to reality. Furthermore, this larger mass-ratio adds an extra challenge to the frequency identification of the bridge model in the experiments as it alters the combined frequency of the car-bridge system.

### 3.3. Instruments

To collect the acceleration data from the robot car, one smartphone, Samsung Galaxy S8, is attached to the top plate, as seen in Figure 8. The acceleration data is recorded with 400 Hz sampling frequency by using an android app previously developed and used in [30], which records the global vertical acceleration by using the accelerometer of the smartphone in combination with the gyroscope and the magnetometer. This app also applies sampling frequency correction to the recorded data, which is not considered in most similar commercial apps. In order to have a benchmark for comparison, one G-Link©-200 wireless accelerometer is connected to the top plate beside the smartphone, as seen in Figure 8, and used to record the acceleration with 512 Hz sampling frequency.

## 4. Analysis

**Figure 10:**
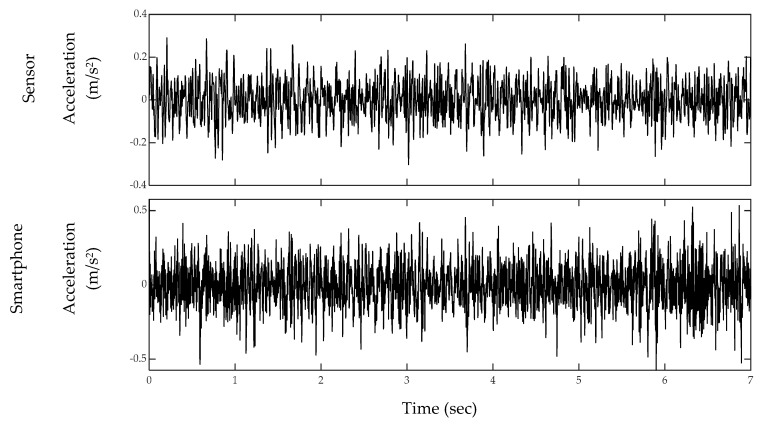
Acceleration signals recorded using the sensor and the smartphone during the off-bridge test.

In this section, the proposed inverse filtering-based method is applied to the data collected in the experiments. First, the off-bridge data is collected and the inverse filter prototype is developed for each case. Later, the filter is verified by applying it to the off-bridge data. Finally, the filter is applied to the on-bridge data in order to identify the fundamental bridge frequency. Note that car speeds of 0.2, 0.3, and 0.4 m/s with the spring stiffness values of 425, 615, and 726 N/m are employed in the experiments, resulting in total nine cases. Besides, since the lengths of recorded acceleration signals vary between 4 to 8 s, a 3 s Hamming window with 75% overlap is considered in ADFT process. Furthermore, to increase the frequency resolution of the ADFT spectrum, all windows are zero-padded to 10 s, resulting in a 0.1 Hz resolution in the ADFT spectrum. In addition, the acceleration signals from the smartphone are resampled with the similar 400 Hz frequency using interpolation before applying ADFT. Resampling provides consistent sampling intervals between recorded data points since the raw data recorded on smartphones are not perfectly consistent and the intervals deviate from the pre-set value. Although the android application developed in the authors’ research group [30] significantly improves the stability of sampling frequency, resampling provides more accuracy in calculations. One sample of the recorded acceleration signals using the sensor and the smartphone are presented in Figure 10. These signals are recorded during the off-bridge test which will be discussed in the next section. As seen, there are some differences between the sensor and the smartphone recorded signals which are due to the placement of the accelerometer inside the smartphone and the vibration of the whole device. However, as will be discussed later, the proposed inverse filtering technique is robust against device features.

### 4.1. Filter Design

**Figure 11:**
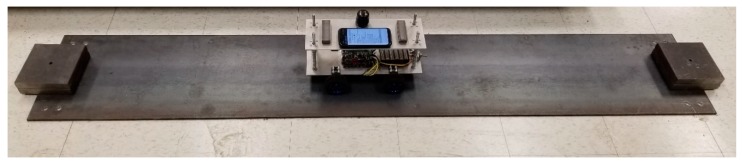
Set-up for simulating the off-bridge condition.

**Figure 12:**
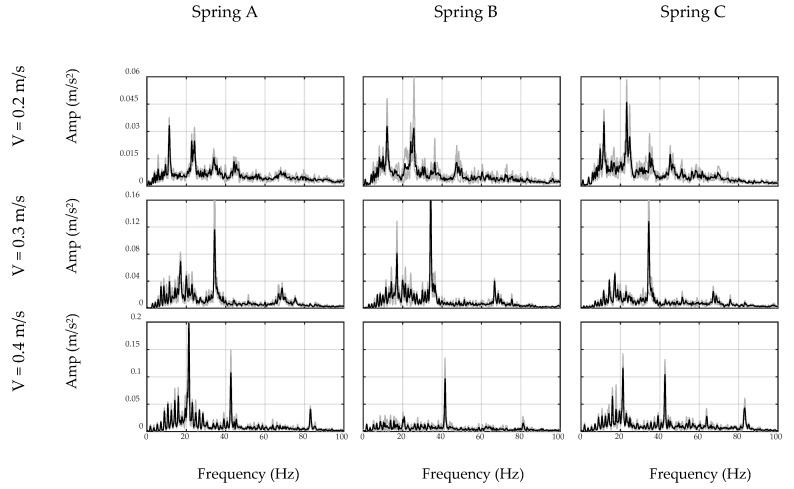
ADFT spectrums of off-bridge signals recorded by sensor.

**Figure 13:**
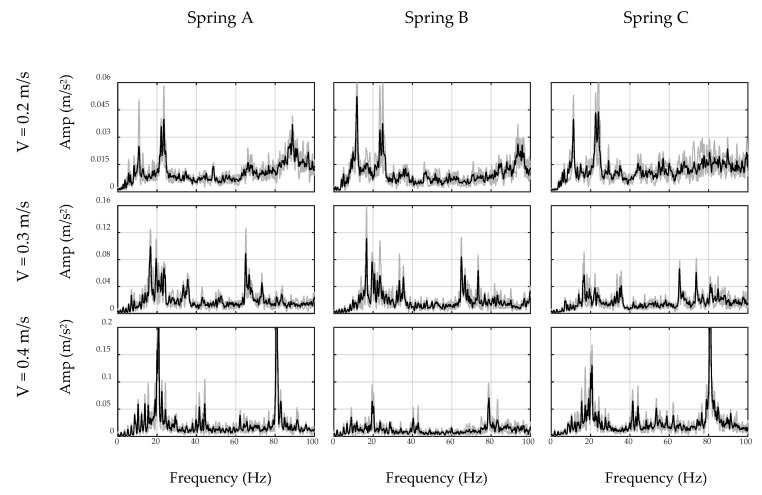
ADFT spectrums of off-bridge signals recorded by smartphone.

First, the car moves on the ground to simulate the off-bridge condition. To this end, a 2 m steel plate similar to the steel deck was put on the ground to mimic the surface condition of the bridge. Since the plate may still vibrate due to imperfections of the floor surface, two masses were used at both ends of the plate to restrain it from any flexural vibration, as seen in Figure 11. The total duration of the off-bridge signal varied between 4 to 8 s for the three speed cases. Therefore, in order to reduce the local effects of the beam and account for longer duration signals, three trials of off-bridge data were collected in this part. ADFT spectrums of the acceleration data recorded by sensor and smartphone are shown in Figure 12 and Figure 13, respectively. These figures consist of three rows and three columns representing different speeds and suspension stiffness values. In each plot, trials are illustrated using gray curves and their average is shown with the black curve. As seen, major peaks which are mainly due to motor vibration and moving frequency of the car are mostly dependent on the speed of the car. In addition, an increase in the speed results in higher amplitude for major frequencies. As expected, the data recorded on the sensor show higher resolution with respect to the smartphone due to the higher accuracy of the device, resulting in capturing low-frequency harmonics with higher accuracy. However, the smartphone is successful in capturing major peaks, which are the focus of the proposed method. In fact, the designed filter for each device will be based on the spectrum of that device and thus will be unique.

**Figure 14:**
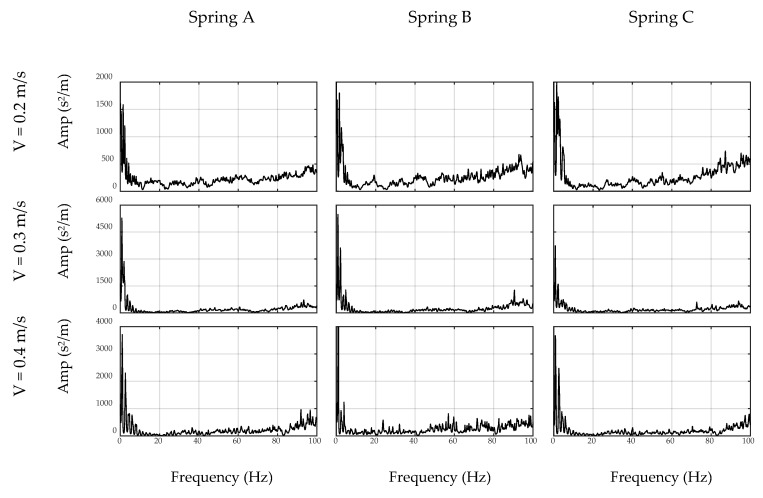
Inverse filter prototype based on collected signals by sensor.

**Figure 15:**
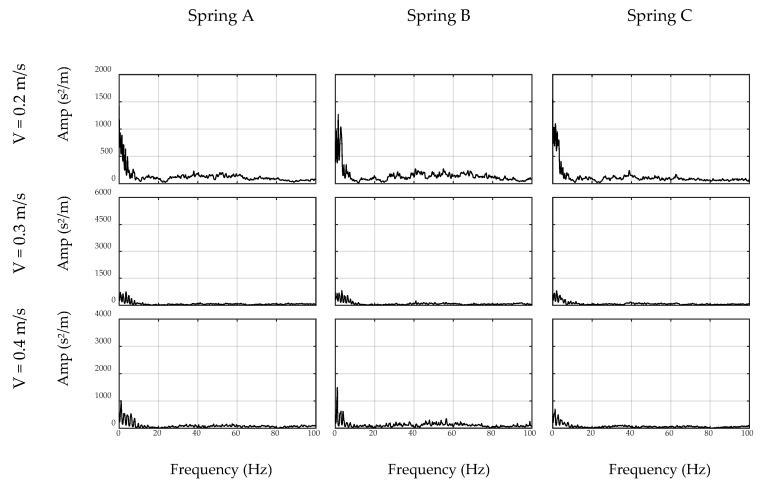
Inverse filter prototype based on collected signals by smartphone.

**Figure 16:**
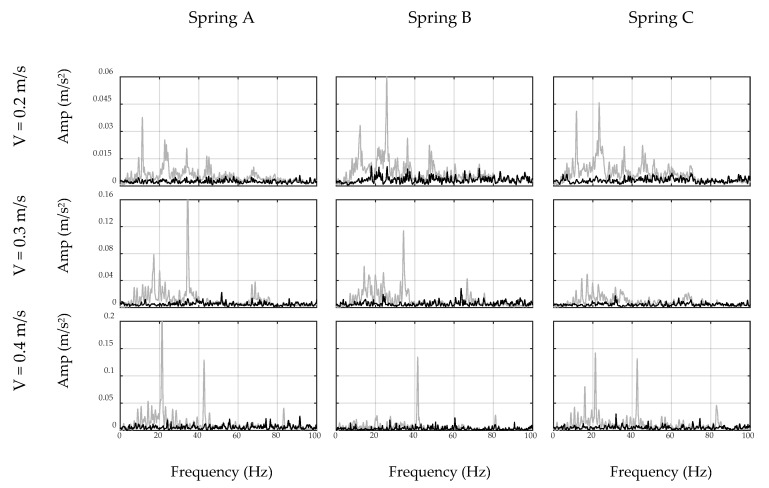
Unfiltered (gray) and filtered (black) spectrums of off-bridge data collected by sensor.

**Figure 17:**
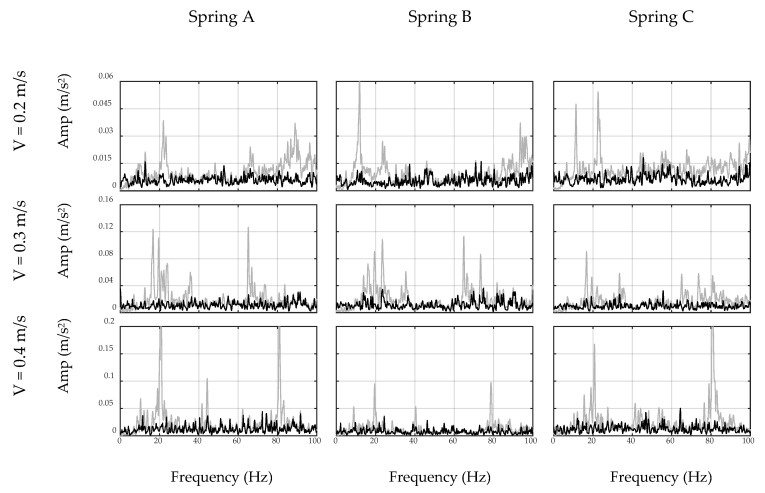
Unfiltered (gray) and filtered (black) spectrums of off-bridge data collected by smartphone.

As explained in the preceding sections, the off-bridge spectrum is used to form the inverse filter prototype, which is illustrated in Figure 12 and Figure 13 for the sensor and smartphone data, respectively. As seen, filter shapes are amplifying low-amplitude frequencies in off-bridge spectrums and suppressing those of high-amplitude. However, as illustrated in Figure 14 and Figure 15, filter shapes of sensor and smartphone have major differences which makes it impossible to use a general filter for all devices even in a similar car. In fact, this is one of the major advantages of the proposed method that considers each device in each car as a separate data source and creates a unique filter for it. In addition, it can be seen that the order of the amplitudes of the filter prototypes, i.e., *y*-axis values in Figure 14 and Figure 15, are inverse of the order of off-bridge spectrum amplitudes, i.e., *y*-axis values in Figure 16 and Figure 17. Thus, it seems reasonable to scale the filter before applying to the on-bridge spectrum to avoid numerical problems.

### 4.2. Filter Verification

Before applying the inverse filter to the on-bridge data, the performance of the filter is verified by applying it to unseen off-bridge data, i.e., new off-bridge data that was not used in the filter development phase. It is expected that the designed filter should remove all major car-related frequencies from the spectrum and no major preference should be present between the frequencies in the filtered off-bridge spectrum. To this end, unfiltered and filtered spectrums of off-bridge acceleration signals are shown in Figure 16 and Figure 17 for the sensor and smartphone data, respectively. In these figures, the original unfiltered spectrums are illustrated by gray curves and the filtered ones are in black. As seen, the filtered data are almost considered as the white noise with no major peaks, which verifies the successful performance of the inverse filter in removing car-related frequency content. In addition, the performance of the filter is stable for all combinations of speed and suspension stiffness which proves the robustness of the proposed method. As expected, the performance of the filter is stronger using the sensor and filtered spectrums have lower variations. However, Figure 17 demonstrates that the filter designed by smartphone-collected data is capable of eliminating most of the major car-related frequency content, which would be used to detect any external frequency content when another vibration source, specifically bridge data, is added to the signal.

### 4.3. Filter Application

**Figure 18:**
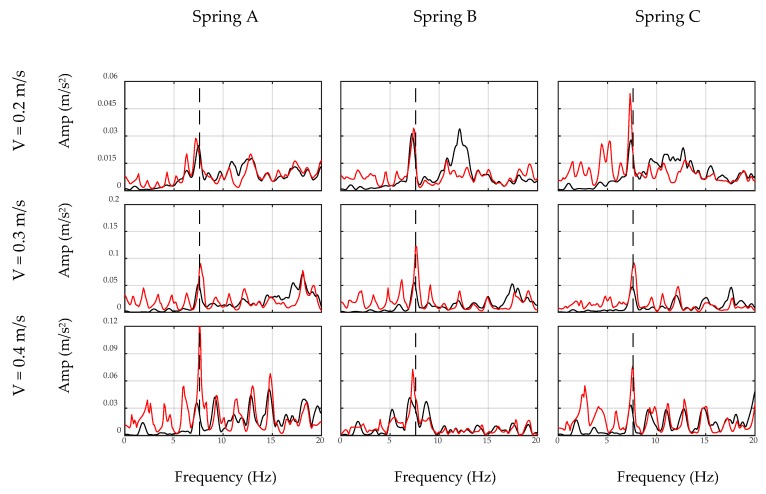
Unfiltered (black) and filtered (red) spectrums of on-bridge data collected by sensor.

**Figure 19:**
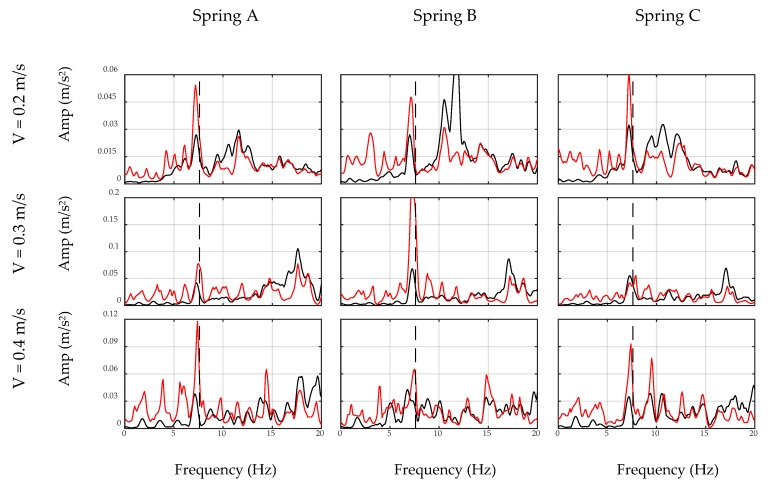
Unfiltered (black) and filtered (red) spectrums of on-bridge data collected by smartphone.

In this section, the car moves over the bridge and the developed inverse filters are applied to the collected acceleration signals, i.e., on-bridge signals. Figure 18 shows the unfiltered and filtered spectrums of on-bridge signals in black and red curves, respectively, for sensor-collected data, while Figure 19 shows the same for the smartphone data. In these figures, a frequency range of 0–20 Hz are considered to focus on the fundamental frequency of the bridge. In addition, the fundamental frequency of the bridge is marked with black dashed lines in both figures. This frequency was identified by a separate test through applying initial displacement at the center of the bridge and recording and analyzing the responding free vibration of the bridge. As seen, filtered spectrums, shown with red curves, amplify the fundamental frequency of the bridge, while detecting the frequency of the bridge among all other peaks is challenging through unfiltered spectrums, shown with black curves. In addition, comparing Figure 18 and Figure 19 demonstrates that the smartphone with lower accuracy performs similarly in detecting the fundamental frequency of the bridge comparing to the sensor. The reason is that the inverse filter was designed based on the performance of the device and hence eliminated device errors. Furthermore, considering different combinations of speed and suspension stiffness evidences the robustness of the proposed methodology against car features. Since the duration of the signals was relatively short in this experiment, i.e., 4–6 s, recorded data may not be able to model the general pattern of the spectrum. It is expected that in real-life conditions, longer signals may improve the performance of the ADFT spectrum and the resulting inverse filter. However, it is also acknowledged that more challenges will be added to the problem in such real-life applications, e.g., surface roughness or speed changes between off-bridge and on-bridge conditions, which are not in the scope of this paper.

The experimental results illustrated in Figure 18 and Figure 19 provide further insight toward indirect monitoring of bridges using frequency analysis of the passing vehicle. For instance, lower speeds provide longer recorded signals, which results in an enriched spectrum with more bridge-vehicle content than higher speeds. Comparing each row of Figure 18 and Figure 19 shows that the presence of the bridge frequency in the spectrums are stronger at the first rows for lower speeds, while at higher speeds, especially third rows, bridge frequency presence is faded. Furthermore, the accuracy of the sensor in recording acceleration signals yields closer frequencies to the exact bridge frequency as seen in Figure 18. However, smartphone results show more shifted frequencies in Figure 19, which could be improved in future studies. In addition, it should be noted that there are small harmonic peaks emerged in the filtered spectrums of both the sensor and the smartphone, which are due to the inconsistency in the speed of the robot car among off-bridge and on-bridge conditions. The speed of the robot car in this experiment is controlled through the voltage of the motor and the voltage value is kept constant during each experiment, which would warrant constant speed on a flat surface. However, the deformation of the bridge deck affects the speed of the robot car, unlike the off-bridge condition where the surface is perfectly flat. Since the current methodology does not account for the effect of the speed change of the car, harmonics of the altered speed emerge as new peaks in the filtered spectrums, even though they are not significant enough to shadow the bridge frequency.

**Figure 20:**
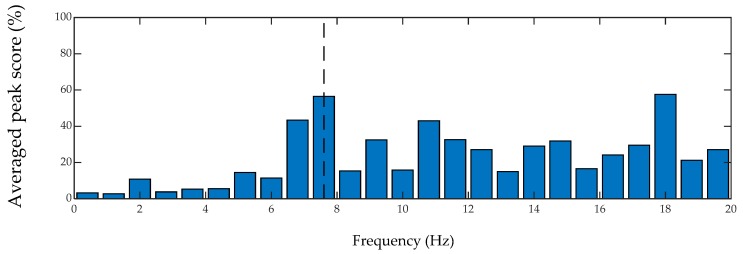
Histogram of averaged peak scores of unfiltered on-bridge spectrums.

**Figure 21:**
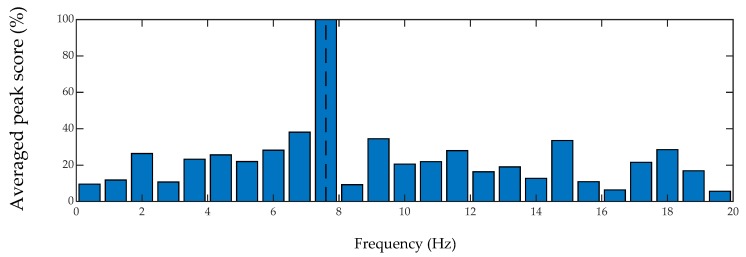
Histogram of averaged peak scores of filtered on-bridge spectrums.

In order to quantify the performance of inverse filtering method on bridge frequency identification, a peak scoring analysis is conducted based on amplitude and prominence using MATLAB. The prominence of a peak is defined to measure how the peak stands out with respect to other adjacent peaks considering its intrinsic height and location. The prominence of an isolated peak with a low amplitude may be higher than another peak that has a larger amplitude but is among a range of tall peaks. On the other hand, two peaks with similar prominence values but with a notable amplitude difference are not equally significant in a spectrum. Hence, both the prominence and amplitude are considered in the scoring criterion of this study. For each spectrum, the prominence and amplitude scores of all peaks are scaled out of 100. Then, the scores are averaged among all passes of the car over the bridge. For simplicity, the smartphone data are considered for peak analysis. The histogram plots of the peak analysis are provided in Figure 20 and Figure 21 for unfiltered and filtered spectrums, respectively. In both plots, the fundamental frequency of the bridge is shown with vertical dashed lines. As seen, considering different cars passing over the bridge at different speeds, the filter can almost double the chance of detecting the frequency of the bridge correctly. In fact, for the performed lab experiments in this paper, 100% of the time, the peak with the maximum amplitude and the maximum prominence was the fundamental bridge frequency. It should be noted that due to the moving frequency of the bridge and also the effect of the car mass in this experiment, the frequency of the bridge is shifted, and the amount of the shift and its sources are not in the scope of this paper.

## 5. Conclusions

This paper proposes a methodology for identifying the fundamental frequency of a bridge by using acceleration signals recorded on cars passing over the bridge. While most studies focus on processing on-bridge data alone, this study suggests the application of off-bridge signals to filter the on-bridge data. The spectrum of the off-bridge data is used to design a specific inverse filter which then could be applied to the on-bridge data. This way, the frequency content of the car is expected to be removed from the on-bridge data, leaving those of the bridge. In order to verify the performance of the methodology, a lab-scale experiment—including a robot car and a simply-supported bridge—was performed considering nine combinations of speed and suspension stiffness values. The main highlights and outcomes of this paper can be listed as: (1) for the first time in the literature, an inverse filtering-based method for indirect frequency identification of bridges is developed; (2) the experiment results demonstrate that inverse filtering provides promising results in suppressing car-related frequencies and amplifying bridge frequency; (3) the filter is designed for each device in each car separately and thus is robust against their features and there is no need to consider the properties of the car or data-collecting devices; (4) the results show that although smartphones have relatively lower accuracy than standalone accelerometers, the proposed inverse filter is able to overcome such challenges by designing unique filter based on the accuracy of the device.

It is expected that as long as the fundamental frequency of the bridge is not close to major frequencies of the car, the filter would be able to successfully extract the frequency of the bridge. In a real-life situation with a variety of cars with different frequencies passing over the bridge, this method is expected to be practical once employed to a large crowdsourced data. Furthermore, the proposed inverse filtering approach is efficient when the speed of the car and the surface roughness level are similar in the off-bridge and on-bridge conditions. Both factors significantly affect the pattern of the acceleration spectrum recorded on the car and their effect should be investigated in future studies in order to achieve a general inverse filtering technique.
